# The Arrestin Fold: Variations on a Theme

**DOI:** 10.2174/138920209787847014

**Published:** 2009-04

**Authors:** Laurence Aubry, Dorian Guetta, Gérard Klein

**Affiliations:** 1 CNRS, UMR 5092, 17 rue des Martyrs, Grenoble, 38054, France; 2 CEA, iRTSV, LBBSI, 38054 Grenoble, France; 3 Université Joseph Fourier, 38000 Grenoble, France

**Keywords:** Arrestins, Vps26, GPCR, retromer, trafficking, endocytosis.

## Abstract

Endocytosis of ligand-activated plasma membrane receptors has been shown to contribute to the regulation of their downstream signaling. β-arrestins interact with the phosphorylated tail of activated receptors and act as scaffolds for the recruitment of adaptor proteins and clathrin, that constitute the machinery used for receptor endocytosis. Visual- and β-arrestins have a two-lobe, immunoglobulin-like, β-strand sandwich structure. The recent resolution of the crystal structure of VPS26, one of the retromer subunits, unexpectedly evidences an arrestin fold in this protein, which is otherwise unrelated to arrestins. From a functional point of view, VPS26 is involved in the retrograde transport of the mannose 6-P receptor from the endosomes to the trans-Golgi network. In addition to the group of genuine arrestins and Vps26, mammalian cells harbor a vast repertoire of proteins that are related to arrestins on the basis of their PFAM Nter and Cter arrestin- domains, which are named **A**rrestin **D**omain- **C**ontaining proteins (ADCs). The biological role of ADC proteins is still poorly understood. The three subfamilies have been merged into an arrestin-related protein clan.

This paper provides an overall analysis of arrestin clan proteins. The structures and functions of members of the subfamilies are reviewed in mammals and model organisms such as *Drosophila*, *Caenorhabditis*, *Saccharomyces *and *Dictyostelium.*

## TUNING OF RECEPTOR-DEPENDENT SIGNALING BY ARRESTINS

In 1985, visual arrestin, also known as S-antigen or arrestin 1, was discovered in bovine retinal rods, where it controls light-dependent cGMP phosphodiesterase activity [1, 2]. Four mammalian arrestins have now been characterized. These are found either specifically in visual systems (arrestin 1 and arrestin 4 in rods and cones, respectively) or ubiquitously (β-arrestins 1 and 2) (for recent reviews, see [3-6]). They comprise a family of proteins that interact with ligand-activated G-protein coupled receptors (GPCRs). The signaling downstream from the receptors is regulated as a consequence of this interaction, and through the interference of arrestins with the subunits of the heterotrimeric G-proteins. This signaling can be fine-tuned by the internalization of GPCRs in coated vesicles formed by the recruitment of clathrin and adaptor proteins by  β-arrestins. GPCR internalization allows either a transient desensitization, when the receptor is rapidly recycled back to the plasma membrane, or a long term desensitization, when targeted to lysosomes for degradation. Additional roles for β-arrestins have recently been unveiled, both with cytosolic partners (interaction with MAP kinases) and in the nucleus (interaction with transcription factors).

Given the way in which GPCRs interact with arrestins (GPCRs respond to a variety of stimuli including hormonal signals, neurotransmitters, chemokines, chemoattractants and sensory stimuli such as light, odor or taste), the size of the GPCR family (over 1000 different GPCRs in humans) and the novel roles that have been uncovered for arrestins, it would appear that we are just scratching the surface of the potential involvement of the arrestin family in a rapidly expanding list of signaling pathways.

## ARRESTINS AND THEIR PARTNERS AS DISCLOSED FROM STRUCTURAL DATA

**Fig. (1). Residue conservation among members of the arrestin family. Localization of binding sites on β -arrestins.**
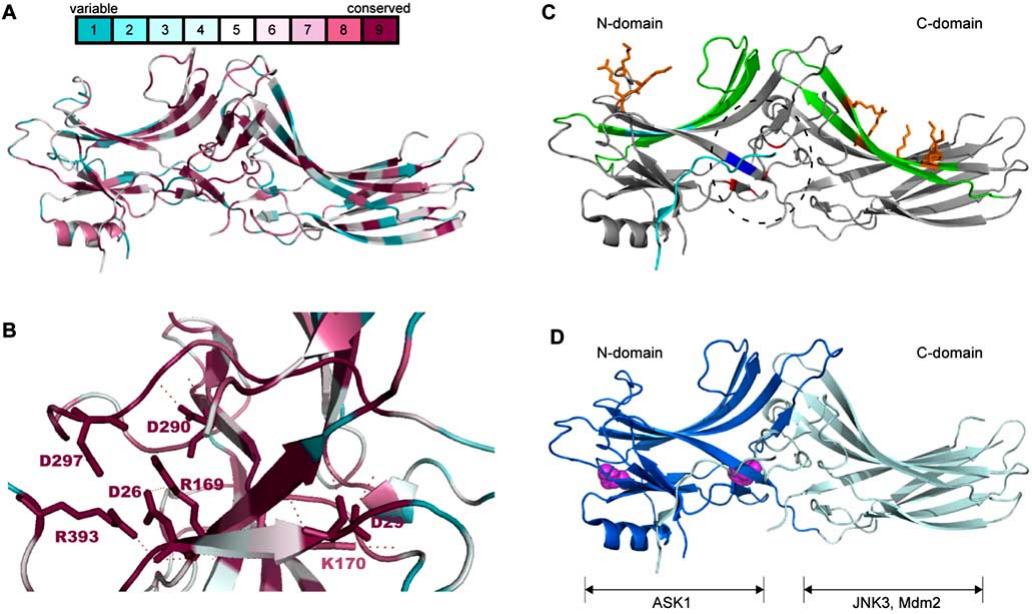
**A.** Amino acid conservation in human rod and cone arrestins and β-arrestins 1 and 2 was calculated with the online Consurf software (http://consurf.tau.ac.il) and represented with PyMol using Consurf coloring (see scale) on β-arrestin 1 template (1ZSH). **B.** Blowup of the polar core showing the high conservation of the residues important for the polar core highlighted as sticks. Electrostatic bonds between core residues are indicated as dotted lines. **C.** The structure of β-arrestin 1 (PDB 1ZSH) is represented with the PyMol software. The localization of the polar core is indicated by a dashed ellipse with positive residues in blue and negative residues in red. Receptor binding β-strands are indicated in green. The adaptin AP-2 binding site is indicated in cyan. Low- and high-affinity binding sites for InsP_6_ in the N- and C-domains, respectively are indicated as orange sticks. **D.** The N- and C-domains of β-arrestin 2 (PDB 1JSY) are indicated in dark and light blue, respectively. Prolines involved in Src binding appear as magenta spheres. The binding domains of ASK1, JNK3 and Mdm2 are indicated.

The crystal structure of visual arrestins and β-arrestins has been determined [7-12]. Both proteins comprise two modules that are related by a pseudo two-fold rotation axis, the N- and C-domains (PFAM signatures PF00339 and PF02752, respectively), which are connected by a flexible loop (Fig. **1A**). Each module, based on the type III module (Fn3) originally discovered in fibronectin and later found in IgGs [13], consists of a deeply curved sandwich of two antiparallel β-sheets. Interestingly, this conserved structure is obtained without any phylogenetic conservation of amino acids. Whether this results from a convergent evolution towards a stable β-sandwich fold or from a common ancestor has not been established.

The C-domain is extended with a flexible C-terminal tail that is buried through interactions with the N-domain and becomes exposed when the arrestin switches from a basal to an activated state. Charged residues from the C-tail, the N-domain and a lariat loop of the C-domain generate a neutral polar core by bridging side chains of opposite charge. This polar core is a critical element for the functioning of arrestins, as it acts as a phosphate sensor and an activation switch (Fig. **1B**): the binding of the negatively-charged phosphorylated C-terminus of activated GPCRs disrupts the electrostatic interactions within the polar core and triggers a conformational rearrangement of arrestin and the subsequent unmasking of the C-tail. It is at the level of this tail that the two arrestin groups differ. A short stretch in the  β-arrestin tail contains clathrin-binding (L[L/I][D/E/N][L/F][D/E], [14, 15]) and adaptin-binding (IVFxxFxRxR, [16, 17]) sites. These sites drive the recruitment of the endocytic machinery and the internalization of the arrestin-bound receptors in clathrin-coated vesicles. Other proteins involved in endocytosis are also scaffolded by arrestins, the monomeric G protein Arf6 and its GEF (ARNO) and GAP (GIT) [18-21]. It has now been demonstrated that β-arrestin 2 serves as a scaffold for endothetial NO synthase that sequentially S-nitrosylates β-arrestin 2 and β2-adrenergic receptor. The consequences of this are the potentiation of the interaction between β-arrestin 2 and clathrin and adaptor protein AP-2, and receptor internalization [22]. Although visual arrestins that regulate the opsin/rhodopsin family of GPCRs do not contain any identifiable clathrin and AP-2 binding sites in their C-terminal tail, visual arrestins Arr1 and Arr2 from the fruit fly promote clathrin-dependent endocytosis of their rhodopsin in physiological or hyperstimulation conditions [23, 24].

Visual and β-arrestins display apparent selectivity towards their respective physiological receptor(s). However, the existence of a biunivocal correspondence between β-arrestins and receptors cannot be envisioned, as the mammalian genome harbors close to a thousand GPCRs and only a few arrestin proteins. The current hypothesis is that the flexibility of the β-arrestin N-domain helps to accomodate the diverse GPCRs, whereas a rigid visual arrestin N-domain best fits its specialized partner, rhodopsin [8]. This is a matter for debate, as class B GPCRs (see definition below) are able to interact with visual arrestin [25], and cone arrestin can bind non-visual receptors in heterologous expression situations [12]. The affinity of GPCRs for β-arrestins and the duration of this interaction in internalization/recycling vesicles is the basis for dividing receptors into two classes. Class A receptors (including the β2-adrenergic receptor, μ-opioid receptor and dopamine receptor) are internalized with β-arrestin 2 preferentially, but rapidly lose β-arrestin binding in the early phase of endocytosis, allowing their rapid recycling and resensitization. Class B receptors (including the V2-vasopressin receptor and angiotensin receptor) bind β-arrestin 1 and β-arrestin 2 equally well, maintain a stable association with β-arrestins on the endosomes and recycle slowly. The information that determines whether GPCRs are classified into class A or B is contained in their cytosolic tail. Swapping of the C-terminus of a class A with that of a class B receptor, or vice-versa, converts the GPCR class [26]. The formation of a stable receptor-β-arrestin complex is dependent upon the presence of phospho-serine and -threonine clusters [27]. The detection of the GPCR phosphorylated state by arrestins is mediated by the polar core, and an arrestin mutated by charge inversion in this core binds non-phosphorylated and phosphorylated receptors equally well [28, 29]. The conformational changes in arrestins that are induced by the sensing of the phosphate moiety allow receptor-binding on surfaces located on the concave side of both the N- (β-strands 5 and 6) and C-domains (β-strands 15 and 16) for both visual arrestin and β-arrestin 1 [30] (Fig. **1C**).

It has been proposed that visual and β-arrestin genes from vertebrates arose from gene duplication of an ancestral gene. As Cnidaria is the oldest eumetazoan phylum [31], arrestin genes from the primitive chordate *Ciona intestinalis* [32] or the Cnidaria *Hydra* *magnipapillata* [33] may be closest to the original arrestin gene.

Phosphoinositols and phosphoinositides appear to be major regulators of visual and non-visual arrestins in their inactive and active states, respectively. Phytic acid (inositol hexakisphosphate, InsP_6_) regulates the oligomerization state of the arrestins, their subcellular location and their interaction with receptors [10, 34, 35]. The concentration of InsP_6_ in cell lines and tissues is generally high, within the 10-100 µM range. Taking into account the affinity of β-arrestin for InsP_6_ of between 50 nM and 1 µM, suggests that cytosolic basal-state arrestin is mostly stored in an oligomeric InsP_6_-bound form. Non-visual arrestins bind InsP_6 _in two low- and high -affinity sites that are located in the N- and C-domains of the arrestins, respectively (Fig. **1C**). Both InsP_6 _binding sites are defined by positive residues patches [10, 11]. The high-affinity InsP_6 _binding residues (K^233^, R^237^, K^251^ in human β-arrestin 2) are located on the concave surface of the C-domain, also interacting with the phosphorylated GPCR [11]. The possibility that the arrestin-GPCR interaction is modulated by InsP_6 _can thus be envisioned. The C-domain site is also able to bind membrane-embedded phosphoinositides, with the highest affinities for PtdIns(4,5)P_2_ and PtdIns(3,4,5)P_3_. Mutations in this binding site impair β2-adrenergic receptor endocytosis [36]. Phosphoinositides may act as co-receptors for coincidence detection, increasing the binding affinity/selectivity of activated arrestins for a phosphorylated GPCR.

## EMERGING AREAS IN β-ARRESTIN SIGNALING

### Extending the List of Arrestin-Regulated Cargoes

Data from different biological models now demonstrate that the role of arrestins is not restricted to serpentine receptors [37, 38], but includes single transmembrane-domain receptors with or without tyrosine kinase activity (type III TGF-β receptor, insulin-like growth factor I receptor, Notch and inhibitory killer-cell immunoglobulin-like KIR receptor), 12-membrane spanning transporter (Na^+^/H^+^ exchanger NHE5) or voltage-dependent cation channel (calcium channel Ca_v_1.2) [39-45]. Three elements in the concave N- and C-domains of β-arrestin 2 interact with NHE5 [41]. Interestingly, the first binding site (residues 52-78) corresponds to the conserved β-strand 5 required for GPCR binding [30]. Whether phosphorylation of NHE5 is a prerequisite for β-arrestin binding has not yet been fully elucidated. In natural killer cells, β-arrestin 2 interacts with KIR engaged with ligands. Interaction necessitates the tyrosine phosphorylation of consensus immunoreceptor tyrosine-based inhibition motifs (ITIM) in KIR’s cytoplasmic tail. This interaction facilitates the recruitment of SHP-1 and -2 phosphatases into a ternary complex that transduces an inhibitory signal abrogating the signal arising from activating receptors [45, 46].

### Extending the List of Arrestin Sites of Action

Recent literature indicates that arrestins are not solely used to squelch receptor-dependent signaling at the plasma membrane. Their role is increasingly comparable to that of heterotrimeric G-proteins, as they act as a secondary signaling platform on the endosomes. Thus they bind c-Src, various MAPKs, PI3K and Akt as well as non-kinase partners such as the cytoskeletal component RhoA (Fig. **1D**) [11]. In the cytosol, β-arrestins also interact with MDM2 and IκBα, and thereby indirectly regulate p53- and NFκB-dependent transcription [47]. The nuclear functions of arrestins have also been documented recently [3]. Both β-arrestins translocate to the nucleus and interact with transcription factors p300 and cAMP-response element-binding protein (CREB) [47]. Furthermore, β-arrestin 1 negatively regulates the transcription factor STAT1 (signal transducer and activator of transcription 1). When the antiviral cytokine interferon–γ binds its receptor, it activates the phosphorylation of STAT1, inducing its dimerization, translocation to the nucleus and transcriptional activity. β-arrestin 1 promotes the dephosphorylation of STAT1 by recruiting the tyrosine phosphatase TC45 in a ternary complex with STAT1, thereby turning off STAT1 and reducing interferon–γ-induced gene transcription. It should be noted that this effect is not observed with β-arrestin 2, though it can bind STAT1, possibly due to a difference in its nucleocytoplasmic shuttling [48, 49]. Indeed, the presence of a functional NES in the C-domain of β-arrestin 2 (^388^FEDFARLRL^396^ sequence), which is absent in β-arrestin 1, prevents its nuclear accumulation. This nucleocytoplasmic shuttling leads to the cytosolic location of c-Jun N-terminal kinase 3 (JNK3) when the kinase is docked on the ^196^RRSLHL^201^ sequence in the C-terminus of β-arrestin 2 [50].

β-arrestin 2, and not β-arrestin 1, is located at the centrosome in both interphasic and mitotic cells, but is not involved in the classical functions of the centrosome, such as nucleation and anchoring of microtubules. Lack of β-arrestin 2 correlates with a reduction in primary cilium formation and cell cycle deregulation [51]. Upon primary cilium formation in fibroblasts, β-arrestin 2 is translocated to the axoneme [51]. Its role there is connected with the Smoothened serpentine protein (Smo) in the Hedgehog (Hh) pathway. Smo is inhibited by Patched (Ptc), a 12 transmembrane-spanning protein. Binding of Hh to Ptc relieves the inhibition of Smo, and this event constitutes the initiation step of the developmental processes. Phosphorylation of Smo by GRK2 induces the recruitment of β-arrestin 2 and the endocytosis of Smo in clathrin-coated vesicles [52]. Once activated, Smo regulates the expression of Hh target genes by controlling the activity of the Glioma-associated oncogene homologue (Gli) [53]. It has now been demonstrated that β-arrestins 1 and -2 promote the interaction of activated Smo with the kinesin motor protein Kif3A, the subsequent movement of Smo into the primary cilium mediated by components of the intracellular transport complex, the production of the activator form of the Gli transcription factor and gene expression [54, 55].

## THE ARRESTIN FOLD, A WIDELY USED MOLECULAR ARCHITECTURE

### Vps26

**Fig. (2). 3D-structure of human arrestin clan proteins.**
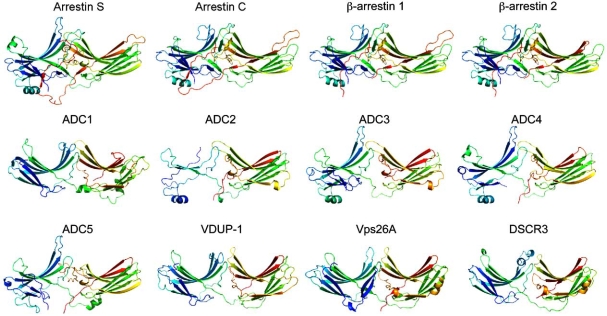
The structure of human arrestin clan proteins was modeled with the Phyre software (http://www.sbg.bio.ic.ac.uk ~phyre/) and visualized with the Pymol software (http://www.pymol.org). The PDB templates used for the modeling were 1CF1 for arrestin S, ADC2, ADC3, ADC4 and ADC5, 1JSY for ß-arrestin 1, ß-arrestin 2 and arrestin C, 2FAU for ADC1, VDUP, Vps26A and DSCR3.

**Fig. (3). Diversity of arrestin clan proteins in human, fruit fly, nematode, yeast and amoeba.**
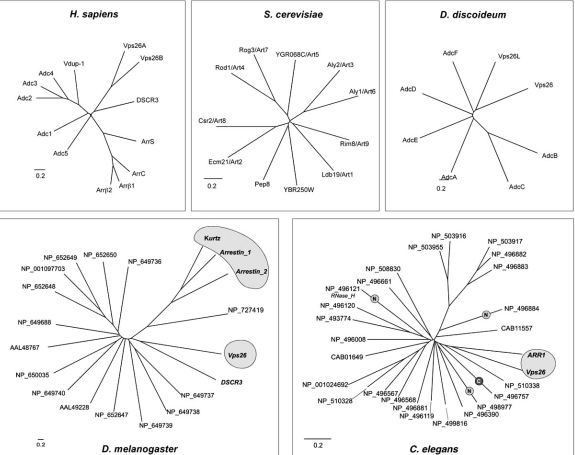
Sequences of human arrestin clan proteins were aligned with ClustalW software [102]. An unrooted phylogenetic tree was drawn with Tree-Dyn for each species (http://www.phylogeny.fr) [103, 104]. Labels in the nematode tree indicate genes in which only the arrestin N- or C-domain was found. A grey cloud highlights proteins in nematode and fruit fly for which literature is available.

Interestingly, the arrestin-fold is also shared by an apparently unrelated protein, Vps26, which is present in humans as two isoforms, Vps26A and Vps26B [56, 57] (Figs. **2** and **3**). Vps26 is a subunit of a large multimeric complex, termed a retromer, which is located on early/recycling endosomes in mammals and which mediates the retrograde transport of the cation-independent mannose 6-phosphate receptor (CI-MPR) and its yeast homologue Vps10 from the endosomes to the Golgi apparatus. The retromer comprises two sub-complexes. The Vps26/Vps29/Vps35 subcomplex is involved in cargo-recognition and loading. The second subcomplex is made up of sorting nexins (SNX) that participate, *via *their PX and BAR domains, in the location of the retromer on PtdIns(3) Prich highly curved membranes of early and recycling endosomes [58-60]. Vps26 interacts with both Vps35 and SNX1.

At present, it is not known whether the structural similarities between Vps26 and the arrestin families correlate with the existence of a functional kinship beyond their role in trafficking processes. In both families, polar and electrostatic contacts play a key role in interdomain interactions. However, when compared to arrestins, the polar core of Vps26 involves different side chains and is generated by distinct secondary structure elements. Mutations of polar core residues in Vps26 do not interfere with either its endosomal location in human cells or with CPY sorting in yeast, thus calling into question its role in the activation step and/or the function of Vps26 [57]. In the retromer complex, the position of Vps26 relative to the neighboring subunits may allow access to the concave surface by target cargoes. So far, however, only the Vps35 subunit has been shown to bind cargoes within the retromer.

### DSCR3

The human genome harbors the DSCR3 gene encoding a protein belonging to the arrestin clan (see definition below) (Fig. **3**). Interestingly, it has been predicted that human DSCR3 contains overlapping PFAM PF00339 (Arrestin, N-terminal domain, E-value 1.9 x 10^-3^) and PFAM PF03643 (Vps26, E-value 1.1 x 10^-155^) domains. Thus, DSCR3 shares sequence kinship with both arrestins and Vps26 proteins and represents an intermediate link between these two families. The full-length structure of DSCR3 can be modeled by homology (Fig. **2**) using the crystal structure of Vps26 (PDB 2fauA) as a template. DSCR3 belongs to the subset of about 20 genes located within locus 21q22, called the **D**own **S**yndrome **C**ritical **R**egion [61], which is involved in the partial or full trisomy of chromosome 21 that is responsible for Down syndrome. Up to now, DSCR3 attracted only a little biochemical interest. Because DSCR3 protein is found in the nucleus, a transcriptional regulation function has been proposed for it [62]. A possible role for DSCR3 and 3 other chromosome 21 trisomy proteins from the DSCR region in a mitogen-activated protein kinase pathway has been predicted on the basis of a computational approach [63].

### Arrestin-Domain Containing Proteins (ADC)

Genes encoding arrestin-domain containing proteins (ADCs) that harbor N and/or C arrestin domain PFAM signatures have been pinpointed in mammalian genomes. Their identity/homology scores with arrestins are, however, very low (11-15% identity with arrestins). In a phylogenic tree (Fig. **3**), ADCs branch away from arrestins, as expected from the poor sequence conservation. The structure of all ADCs can be modeled by homology using the structure of crystalized arrestins (Fig. **2**). It should be noted that the interface between the modeled N- and C-domains does not seem to involve positively and negatively charged amino acids. Data pertaining to the cellular function of these ADCs are scarce. One of them, VDUP-1, is a gene, the mRNA of which increases 4-5 fold upon treatment of HL60 cells with 1,25-dihydroxyvitamin D_3_ [64]. In a yeast two-hybrid screen, VDUP-1 has been shown to interact with thioredoxin [65, 66] and hence to regulate the redox state of the cell [67, 68]. Suppression of thioredoxin activity by VDUP-1 has antiproliferative and apoptotic effects [69]. The expression of ADC2 has been shown to respond to lysergic acid diethylamine in rat and the gene was therefore named *ILAD-1* (induced by lysergic acid diethylamine) [70].

Altogether, visual and β-arrestins, arrestin-domain containing proteins (ADCs) and arrestin-fold proteins (Vps26 + DSCR3) make up a clan of proteins sharing the same structure. Hereafter, they will be referred to as arrestin clan proteins. Alvarez recently suggested that arrestin clan proteins be classified into two subfamilies, the β-class arrestins (visual and β-arrestins) and the α-class arrestins (ADCs), on the basis of the following characteristics: 1) β-arrestins possess a short α-helix in their N domain that is missing in α-arrestins; 2) α-arrestins harbor WW domain-binding motifs (P/L)PxY, but β-arrestins do not. Vps26 proteins are classified as α-arrestin-like [33]. As defined here, these criteria do not seem robust enough to define two subfamilies among human arrestin clan proteins because, firstly, the modeling of ADC2, ADC3, ADC4 predicted by Alvarez as α-arrestins yields a structure of β-arrestins with the characteristic α-helix I in their N domain (Fig. **2**) and secondly, ADC5, defined as a putative α-arrestin on the basis of the absence of helix I, does not harbor any (P/L)PxY motif either.

## VARIATIONS IN OTHER MODELS

Alternative models were studied soon after the discovery of vertebrate arrestins and important roles established in flies or chordates. Data now extend to unicellular eukaryotes.

### Drosophila melanogaster

Visual arrestins 1 and 2 were quickly described in *Drosophila* [71-73] after the discovery of arrestin in bovine retinal rods. Arrestins 1 and 2 are expressed in fruit fly photoreceptor cells. Binding of arrestin 2 to rhodopsin is not dependent on its phosphorylation, whereas arrestin 1 is recruited on phosphorylated rhodopsin. This latter interaction promotes light-induced rhodopsin endocytosis. Arrestin 1 is also required for light-independent photoreceptor survival [24].

More recently, a nonvisual arrestin, Kurtz, has been shown to be an essential neural gene in *Drosophila* [74]. In an arrestin-translocation assay, Kurtz binds amine and various peptide receptors in response to ligand stimulation. Kurtz null mutants are hypersensitive to osmotic stress, involving an as yet unknown GPCR desensitization in the stress response [75]. Kurtz downregulates the Notch cell surface receptor, a single transmembrane protein carrying EGF-like repeats that, together with its ligand Delta, determines the ectodermal or neuronal fate of cells. Kurtz is able to complex Deltex, a E3 ubiquitin ligase. Deltex, in a ternary complex with Notch and Kurtz, will ubiquitinate the intracellular domain of Notch. The outcome of this ubiquitination is the degradation of Notch, possibly by the proteasome [44, 76].

Work on *Drosophila* and on the nematode *Caenorhabditis elegans* (see below) has revealed the essential role of a traffic loop involving Vps26 and the retromer machinery during development. This role is conserved during mouse embryogenesis [77]. Important clues for the understanding of the regulation mechanism of Wg/Wnt secretion by the retromer have now been produced in fruit fly experiments in which Vps26 and Vps35, two subunits of the retromer, are eliminated by RNA-interference. The Wingless (Wg) morphogen is normally carried from the Golgi apparatus to the cell surface by Wntless (Wls), a multi-pass transmembrane protein that acts as a Wg cargo receptor. Once unloaded, Wls is recaptured by endocytosis and recycled to the trans-Golgi network by the retromer complex. In the absence of a functional retromer, Wls is targeted to the lysosome and degraded, thereby preventing Wls to direct Wg/Wnt secretion [78-80].

In addition, the fruit fly repertoire of arrestin clan proteins includes a DSCR3 protein, 14 ADCs and an uncharacterized branch, NP_727419. This gene encodes a long isoform (804 aa) branching off before the divergence of arrestins 1 and 2 and Kurtz (Fig. **3**).

### Caenorhabditis elegans

The repertoire of nematode arrestin clan proteins comprises a single arrestin, a Vps26 protein and a crown of ADC proteins (Fig. **3**). The presence of some minor branches may be due to not yet stable entries in the databases. ARR-1, the sole genuine arrestin encoded by the nematode genome, is highly similar to fruit fly Kurtz. While the disruption of the G protein-coupled receptor kinase 2 (grk-2) gene strongly affects chemotaxis, disruption of ARR-1 does not perturb chemosensation, which is a paradox, since GRKs and arrestins cooperate in the regulation of receptor-dependent signaling [81]. ARR-1 is dispensable in development, but has been shown to regulate olfactory adaptation and recovery. The C-terminal domain harboring a conserved clathrin-binding box (LIQLH) and a β2-adaptin binding box (FxxxR), is essential to promote internalization of the β2-adrenergic receptor when nematode ARR-1 is expressed in HEK293 cells [82].

The first evidence that the retromer is involved in the trafficking of Wntless was provided by studies on *C. elegans.* The retromer functions in the formation of a gradient for EGL-20, the nematode ortholog of Wnt, as disclosed by RNA-mediated interference against *vps35*, *vps29* and *vps26, *and regulates neuronal polarity** [83, 84].

The NP_496121 gene encodes a fusion protein of the N-domain of arrestin and RnaseH. No data are available for the arrestin-domain containing proteins.

### Saccharomyces cerevisiae

Until recently, arrestins were thought to be restricted to metazoa. The fungi *Aspergillus nidulans* and *Saccharomyces cerevisiae* also harbor arrestin-related proteins involved in the response to various stresses involving either pH, chemicals or heavy metals. Eleven candidates are found in *S. cerevisiae*, which is responsible for the **R**esistance of yeast to **o**-**d**initrobenzene (Rod1p and Rog3p), involved in the response to alkaline ambient pH (Rim8/PalF) or cadmium (Ecm21 and Csr2) or interacting with the autophagic machinery (Aly1p and Aly2p) (Fig. **3**) [85-92]. Although not all candidates share the following properties throughout, they generally contain the PFAM N- and C-domains of arrestins and the WW-binding sequences PPXY, criteria that have been proposed for the α-arrestins. These “PY” sites allow yeast arrestins to bind the HECT-type ubiquitin ligase Rsp5 and to be ubiquitinated by it. Phosphorylation of arrestins and of the regulated GPCR(s) is an important regulation step in mammals. Rod-1 is phosphorylated by Snf1-kinase and the kinase activity is important for drug resistance [91]. Aly2p is phosphorylated by Cdk1 [93]. PalF in *A. niger* and Ecm21 in *S. cerevisiae *promote the internalization of the pH sensor PalH or the yeast manganese transporter Smf1, respectively [87, 90].

Three genes loosely related to arrestins complete the repertoire of yeast arrestins: YGR068C, Ldb19 (low dye binding) and YBR250W. YBR250W/Spo23 is a member of the yeast sporulation pathway [94]. The null mutant is viable and has no sporulation defect [94, 95]. All of these genes except YBR250W have been renamed arrestin-related trafficking adaptors (ARTs). ART1 (formerly Ldb19) and ART2 (Ecm21) are used as scaffolds for the recruitment of the ubiquitin ligase Rsp5 in the neighborhood of their target cargoes, a step that is followed by ubiquitination and endocytosis of the cargoes [96]. All yeast ARTs may function as scaffolds for the endocytosis of plasma membrane proteins, in response to environmental stresses.

Pep8 is the genuine yeast Vps26. It has been demonstrated that it forms the retromer complex with Vps35, Vps29, Vps17 and Vps5 [97] and bridges the cargo-binding protein Vps35 and the membrane deformation subcomplex Vps5/Vps17 [98]. Pep8 is essential for the correct sorting of carboxypeptidase Y to the vacuole [99]. The retromer cooperates in endosomal cargo retrieval back to the Golgi apparatus with BTN2, the yeast homolog of mammalian Hook1, through the direct interaction of BTN2 with Vps26 [100].

### Dictyostelium discoideum

The unveiling of PalF and more recently Ecm21, two fungal proteins with arrestin domains and functions, prompted us to screen for whether other unicellular eukaryotes, in particular the amoeba *Dictyostelium discoideum,* code for proteins of the arrestin clan. A total of eight candidates were found in *D. discoideum *(Fig. **3**). Most obviously, hits for Vps26 and DSCR3 were identified on the basis of their strong conservation with human orthologs (1e^-109^ expect value and 55% identity for DDB_G0269168/Vps26; 7e^-94^ expect value and 56% identity for DDB_G0292212/DSCR3) (Fig. **3**). As for the human protein, amoebal DSCR3/Vps26L has a Vps26 PFAM domain that overlaps in its first half with an arrestin C-domain.

**Fig. (4). Domain organization of Dictyostelium arrestin clan proteins.**
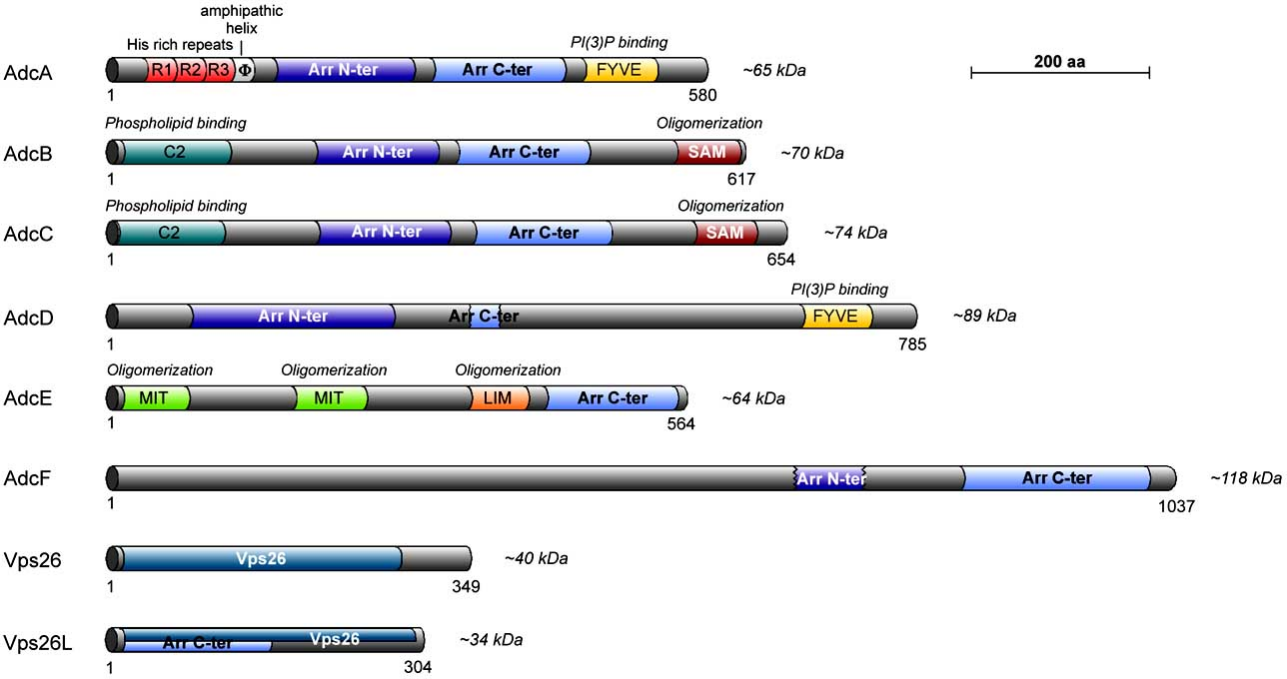
*Dictyostelium* arrestin-domain proteins (Adcs), Vps26 and Vps26L/DSCR3 are represented by their colored domains as indicated. Number of amino acids and molecular masses of each protein are indicated. The functions of each domain are indicated on top.

Besides these 2 proteins, 6 candidates harbor either both arrestin N- and C domains (AdcA, -B, -C, -D, -F) or the C-domain only (AdcE). Interestingly, most arrestin-related proteins from *Dictyostelium* exhibit new pairings of known domains. They are extended on both sides of the arrestin core, with consensus protein-protein interaction domains (SAM, MIT, LIM) or protein-phospholipid interaction domains (FYVE and C2) (Fig. **4**). Such a domain organization is also found in *Entamoeba* *histolytica* arrestins and seems to be limited to Amoebozoae. The presence of C2 and FYVE domains may indicate that ancient arrestins have targeted functions at the plasma membrane and the endosome, respectively. Interestingly, AdcA has also been found in the protein inventory of the centrosome [101] as has β-arrestin 2 in fibroblasts.

AdcB, AdcC and AdcD harbor “PY” sequences outside their arrestin core (AdcA: PPVY; AdcC: LPRY) or within the arrestin N-domain (AdcD: LPKY) as do mammalian α-arrestins and yeast ARTs, which may serve to recruit a HECT domain-containing ubiquitin ligase. The structure of whole AdcA, AdcB and Vps26 has been modeled by homology on the structure of crystalized arrestin S or Vps26A. It shows the general conserved structure of arrestins with an α-helix in the N-domain of AdcA as do visual and non-visual arrestins. Unexpectedly, the modeled structure of AdcA does not exhibit a polar core with opposite-charged residues able to act as the sensor for a phosphorylated receptor, suggesting a different mode of activation.

## CONCLUSION

The arrestin clan comprises proteins, visual and non-visual arrestins, arrestin-domain containing (ADC) proteins, the retromer subunit Vps26 and DSCR3, which share a common structure. In invertebrates, members of all subfamilies can be found with at least a β-arrestin, a visual arrestin when the organism has eyes or ocelli (*Drosophila*,* Ciona*), Vps26 and DSCR3, and a broad representation of ADC proteins. Arrestin clan proteins have also been found in unicellular eukaryotes (yeast and *Dictyostelium*). In most cases where studies have been performed, arrestin clan proteins have been linked to intracellular trafficking, though they belong to different machineries in different cellular locations. This points towards the existence of different functions for proteins built on a conserved structure, in which the 3D-structure is essential for scaffolding the various affiliates.
